# Editorial: *APOE4*-associated heterogeneity in the pathogenesis of Alzheimer's disease

**DOI:** 10.3389/fnagi.2024.1509307

**Published:** 2024-11-19

**Authors:** Eileen Ruth Samson Torres, G. William Rebeck, Tal Nuriel

**Affiliations:** ^1^Department of Neurological Surgery, Weill Cornell Medicine, New York, NY, United States; ^2^Department of Neuroscience, Georgetown University, Washington, DC, United States; ^3^Taub Institute for Research on Alzheimer's Disease and the Aging Brain, Columbia University, New York, NY, United States

**Keywords:** Alzheimer's disease (AD), Apolipoprotein E, *APOE4*, heterogeneity, lipids, high fat diet, estrogen, ARIA

Possession of the Apolipoprotein E ε4 (*APOE4*) allele is the major genetic risk factor for the late-onset form of Alzheimer's disease (AD). While substantial research has been devoted to understanding the mechanisms responsible for the elevated AD risk among *APOE4* carriers, much less research has focused on the specific pathological differences that exist between *APOE4* carriers and non-carriers. This information is crucial, however, as we advance into a new era of AD research and treatment, where understanding these pathological differences may enable clinicians to use targeted strategies for AD prevention, diagnosis, and therapy in *APOE4* carriers vs. non-carriers.

To help elucidate this *APOE4*-associated heterogeneity in AD pathogenesis, we organized a Research Topic devoted to this subject. As described below, the seven articles that were published as part of this Research Topic each help elucidate an important factor related to this *APOE4*-associated heterogeneity. Importantly, these articles also highlight the diverse effects that an individual's *APOE* genotype has on their body and brain during aging and AD pathogenesis.

The primary role of the apoE protein is to aid in the transport and cellular uptake of cholesterol and other lipids within the periphery and the CNS (Yamazaki et al., 2019). Mares et al. utilized human lipidomics and transcriptomics databases from brain tissues and blood samples from the ROSMAP cohort. The authors identified a convergence of differences between cognitively unimpaired *APOE4* carriers and MCI patients, especially in lipids associated with the acyl chain remodeling pathway. This connection implies that membrane composition and lipid signaling related to this pathway are altered early in the process of AD pathogenesis in *APOE4* carriers, occurring even before any clinical symptoms are observed.

ApoE-containing lipoproteins that circulate through the blood are primarily produced by the liver, while those that exist in the brain are primarily produced by astrocytes. While these two apoE lipoprotein pools are physically separated from one another by the blood brain barrier, Han et al. found that liver function in *APOE4* carriers can have a significant impact on the development of AD-related brain pathology. Using two independent cohorts (ADNI and a study at Hallym University Medical Center), the authors reported that liver damage correlated with an increased risk of brain Aβ and AD, but that this connection was unique to *APOE4* carriers. Thus, *APOE4* genetic risk may combine with liver dysfunction risk to drive AD pathogenesis. Furthermore, Guan et al. assessed the effect of *APOE4* carrier status on the interaction between diet, blood biomarkers, and brain white matter integrity in a cohort of 156 individuals of Puerto Rican descent over 12 years. They found that *APOE4* carriers possessed greater associations between numerous baseline dietary factors/blood biomarkers and 12-year medial temporal lobe white matter integrity. On the other hand, while cardiovascular disease and body mass index were also associated with white matter integrity, this association was not affected by *APOE4* carrier status.

Another important aspect of *APOE4*-associated AD risk is the varying disease penetrance that exists between male and female *APOE4* carriers and between *APOE4* carriers with differing races/ethnicities (Belloy et al., 2023; Farrer et al., 1997). For example, it has been reported that female *APOE4* carriers (regardless of racial/ethnic background) possess an increased risk of AD compared to their male counterparts (Belloy et al., 2023). While the reason for this increased AD risk in women has not been fully elucidated, previous studies have uncovered specific metabolic changes that occur in the female brain during menopause as a potential mediating factor (Rahman et al., 2020; Breeze et al., 2024), with several studies reporting that early intervention with hormone replacement therapy can reduce AD risk in women (Nerattini et al., 2023; Coughlan et al., 2023; Saleh et al., 2023). On this topic, Wugalter et al. measured serum estrone (E1) and estradiol (E2) levels in postmenopausal women from the MsBrain cohort to assess the separate and interactive associations of estrogen, plasma AD biomarkers, and *APOE4* carrier status on regional brain volumes. The authors reported that higher endogenous estrogen levels were associated with increased regional brain volumes only in women with more severe AD biomarker profiles. However, this effect was driven by *APOE4* non-carriers, suggesting that *APOE4* carriers do not benefit similarly from higher estrogen levels on regional brain volumes.

Balu et al. also investigated the interaction of *APOE4* carrier status and sex, this time in a mouse model of *APOE* genotype and AD. Utilizing the EFAD mouse model of AD, the authors reported that the combination of *APOE4* and female sex led to earlier cognitive impairment in the mice, as well as increased Aβ pathology and higher levels of neuroinflammation. Interestingly, female *APOE3* EFAD mice possessed similar pathology to male *APOE4* EFAD mice, indicating that female sex alone can lead to greater AD pathology in these mice. Meanwhile, Christensen et al. utilized only female *APOE* mice to explore the relationship between obesity (a modifiable AD risk factor), estrogen therapy, and cognitive deficits. When female *APOE3/3, APOE3/4*, and *APOE4/4* mice were fed a high fat diet (HFD), the *APOE4/4* mice were overall more impaired cognitively and had worse metabolic function; however, the *APOE3/3* mice were more affected by the HFD compared to their regular chow-fed counterparts. Interestingly, estradiol treatment improved metabolic function and cognitive performance in all the HFD-treated mice, but the *APOE4/4* mice benefitted the most.

Lastly, it is important to note that *APOE4* associated heterogeneity is already contributing to real-world AD treatment decisions, since *APOE4* carriers are known to be at greater risk of developing Amyloid-Related Imaging Abnormalities (ARIA) while being treated with the newly approved amyloid immunotherapies Leqembi and Kisunla (Sims et al., 2023; van Dyck et al., 2023). In a review article by Foley and Wilcock, the authors highlighted three hypothesized mechanisms by which *APOE4* may be influencing ARIA risk: (1) reduced cerebrovascular integrity, (2) increased neuroinflammation and immune dysregulation, and (3) elevated levels of cerebral amyloid angiopathy (CAA). Importantly, they detail how these three potential mechanisms could be pursued in clinical and preclinical studies, emphasizing that the usefulness of AD therapies, now and in the future, requires that the AD research community understand the pathological heterogeneity mediated by possession of the *APOE4* allele.
